# Assessment of safety attitudes, professionalism and exploration of medical students’ experiences

**DOI:** 10.1186/s12909-022-03387-7

**Published:** 2022-04-26

**Authors:** Fatemeh Keshmiri, Mehdi Raadabadi

**Affiliations:** 1 Medical Education Department, Education Development Center, Shahid Sadoughi University of Medical Sciences, Yazd, Iran; 2 Health Policy and Management Research Center, School of Public Health, Shahid Sadoughi University of Medical Sciences, Yazd, Iran

**Keywords:** Safety, Attitudes, Professionalism, Medical student, Qualitative, COVID-19 pandemic

## Abstract

**Background:**

The present study was conducted to examine the interns’ perceptions of safety attitude and professionalism and to explore their experiences about adherence to the principles during the COVID-19 pandemic.

**Method:**

The present study was a mixed-method that was performed in two quantitative and qualitative stages. The medical interns at X University (*n* = 140) were entered. In the quantitative phase, the assessment of the interns’ Safety Attitudes and Professionalism was conducted by a survey. In the qualitative phase, data were gathered by semi-structured interviews. The experiences of participants were analyzed by the inductive content analysis approach of Graneheim and Lundman.

**Results:**

Participants’ perception scores on safety attitude and professionalism were 98.02 (14.78). The results were explained in a theme of “weakness in systemic accountability in compliance with professionalism and safety”. The theme included three categories: ‘support system inadequacy’, and ‘null curriculum in safety and professionalism education’.

**Conclusion:**

The present results showed participants’ perception scores on safety attitude and professionalism were below the moderate level. The systemic issues were explored as influencing factors in the occurrence of unsafe and unprofessional behaviors. They reported the weakness of the support system (individual, teamwork, mental health, well-being, management, and culture), and the null curriculum in education of professional, and safety principles effective on unprofessional and unsafe behaviors. During the COVID-19 pandemic, it is recommended to create mechanisms to support the development of professionalism of healthcare workers, especially, novice providers and students, and pay attention to the safety and professionalism in formal and informal educational programs.

## Introduction

During COVID-19 epidemic, the implementation of patient safety approach face challenges of professionalism among healthcare workers [1]. Patient safety was defined processes or structures that reduce adverse events while ensuring safety of medical care and procedures [2]. The growing complexities in the healthcare services have turned the challenge of patient safety and patient-centered services in the global health care systems into a matter of concern [3]. To meet the challenge of safety, compliance with professional and safety principles has been defined as one of the professional and ethical obligations of all healthcare workers and has been highlighted in professionalism guidelines [1, 4]. Stark points out that poor practice in medicine is often caused due to unprofessional behaviors rather than lack of physician’s knowledge or skills [5]. The results of Bahaziq’s review study showed a positive relationship between unprofessional behaviors and adverse outcomes such as endangering the patient’s life, patient dissatisfaction, and medical errors [6]. People who adhere to the best principles of professionalism are expected to pay attention to safety principles [5].

During the Covid-19 pandemic, the issue of safety and compliance with professional principles has acquired more complex dimensions than the provision of routine services and it became a great issues in healthcare systems [1, 7]. The individual protection against professional duties, as well as the respect and confidentiality of patients who not only suffer from the disease but are also carriers of the virus and can infect others, represented a challenge for the fulfillment of professional principles and safety [1].

In the pandemic, in addition to the defects of professional and safe behavior, the challenges of attitudes in the area of professionalism and the dilemma of patient safety concerning self-preservation have increased the occurrence of unprofessional and unsafe behaviors [1, 8, 9]. Dhai et al., showed the concerns raised about the protection of privacy and respect for rights of infected individuals [1]. Sudarmika et al., showed the number of recordings and reporting on patient safety such as the implementation of patient identification, effective communication, the accuracy of surgical procedures prevention of falling risk and infection prevention were decreased before the pandemic and during the pandemic because healthcare providers were more focused on providing the care of patients with Covid-1 9 [9]. The Covid-19 epidemic has severely affected the safety of patients and healthcare personnel and posed a serious threat to both of them [10, 11]. Denning’s study assessed the impact of the Covid-19 pandemic on the patient safety culture of health workers in London that showed improvements in safety attitudes during Covid-19 compared with baseline in allied health professionals and doctors, but no change in nurses [11]. Patient safety and professionalism are context-based concepts that are influenced by a variety of factors [1, 7, 9, 12, 13]. Different results in the studies can be due to differences in safety culture and professionalism in the investigated environments [1, 7, 9, 11–13]. As well, Liao et al., were introduced organizations’ cultural factors such as safety culture, teamwork culture, error disclosure culture, and professionalism are important factors for understanding aspects of the healthcare workplace environment that have been linked with patient safety and quality of care. In Liao’s study was developed the Medical Student Safety Attitudes and Professionalism Survey (MSSAPS) to measure students’ perceptions of these cultural factors in their clinical rotations [14]. In Liao’s study was adapted items from the Safety Attitudes Questionnaire (SAQ) for safety culture and teamwork culture domain, the SAQ and moral distress in clinical learning environments survey for professionalism domain, AHRQ’s hospital survey, and student safety perceptions for patient safety and error disclosure culture items from disclosure for safety culture assessment [14]. In the present study, we used Medical Student Safety Attitudes and Professionalism Survey (MSSAPS) to understand how the cultural factors affect medical students’ perceptions in their clinical rotations were conducted.

The COVID-19 pandemic result in students engaged as part of the workforce and embedded in the clinical environment [15]. Therefore, it is necessary to examine the factors that influence the incidence of unprofessional and unsafe behaviors in viewpoints of them. To the authors’ knowledge, limited studies have been conducted on the effect of epidemics on patient’s safety [9, 13], due to the complexity of patient safety and the influence of various factors at different levels when unprofessional and unsafe behavior occurs, it may be helpful to identify these factors using a mixed-method (quantitative-qualitative) approach. The present study was conducted to examine the interns’ perceptions of safety attitude and professionalism and to explore their experiences about the factors influencing adherence to the principles during the COVID-19 pandemic.

## Method

The present study was a mixed-method that was performed in two quantitative and qualitative stages. According to a mixed-method sequential explanatory design that was proposed by Creswell and Zhang, the quantitative step is the main part of the present research. Firstly, the quantitative step was conducted through a survey to investigate the safety and professionalism perception of the medical interns. Secondly, in the qualitative step was explored the experiences of 16 medical interns to help explain the quantitative results using individual interviews (Table 1).

**Table 1:** Process flow diagram of the procedures for this sequential explanatory mixed methods study. (This table was inspired by Creswell’s (2015) the explanatory sequential mixed method design) [16]

Phase	Procedure	Product
Quantitative data collection	Cross sectional survey	Numeric data
Quantitative data analyses	Use of descriptive and inferential statistics	Meaningful measures
Connecting Quantitative and qualitative phase	Selection pf participants purposefully and interview questions development	Interview guide
qualitative data collection	In-depth individual interview	Transcribing data
qualitative data analyses	Inductive content analysis	Codes, categories and theme
Integration of the quantitative and qualitative results	Interpretation and explanation of the quantitative and qualitative results	Discussion
		Implication
		Further research

### Participants

All medical interns who have spent at least 6 months of internship in the hospital at X University (*n* = 140) were entered by the census. In the qualitative phase, the Maximum-variation sampling method was used and the interns who achieved the highest and lowest perception scores and agreed to participate were purposefully contributed in the phase. In the qualitative phase, 16 medical interns were participated.

#### Quantitative phase

Examining the interns’ Safety Attitudes and Professionalism.

In this phase, the medical interns’ safety and professionalism perception was surveyed. Liao et al. (2014) developed and validated the Medical Student Safety Attitudes and Professionalism Survey (MSSAPS) [14], which was used in this study. The questionnaire was developed in five categories including ‘safety culture’ (*n* = 10),’ teamwork culture’ (*n* = 8), ‘error disclosure culture’ (*n* = 6), ‘experiences with professionalism’ (*n* = 3), and ‘comfort expressing professional concerns’ (*n* = 7). Scoring was a 5-point Likert scale. (1 = disagree strongly to 5 = agree strongly). The score range of the MSSAPS is from 34 to 170. The authors assessed the validation of MSSAPA in the previous study [17]. The validity and reliability of MSSAPS were confirmed (Cronbach’s alpha coefficient = 0.859) in the investigated context. In addition, a confirmatory factor analysis test was approved (CFI, GFI and RMSEA indices were 0.91, 0.92 and 0.08, respectively) [17].

The anonymous questionnaire was distributed among medical interns and collected by the researcher (M.R) in the teaching hospitals.

Data was analyzed by descriptive (frequency, Mean, SD) and analytical (Student t-test, ANOVA, Pearson) tests.

### Qualitative phase: the exploration of the medical interns’ experiences

The inductive content analysis approach as described by Graneheim and Lundman was used in the present phase [18]. Inductive content analysis is a suitable method for investigating new areas in an exploratory manner, or for exploring a known area from a fresh perspective, recognizing the challenge of the safety and professionalism approach from the perspective of medical students can be helpful in identifying the problem and their causes during COVID-19.

### Data collection

The data were collected through semi-structured in-depth individual interviews. Each interview lasted between 45 to 90 min. The time and location of the interview were arranged with the participants prior to the interview. The interviews were held in a quiet location in the teaching hospitals. All of the interviews were conducted by a trained interviewer. The author (M.R.) has significant prior involvement in the field of medical education. Prior to the interview, the aim of the study, the method of the interview, and the participants’ rights to withdraw from the study at any point were explained. We ensured the participants of the confidentiality of the recorded data and obtained informed written consent from all participants. The interviews were directed based on the interview guide that developed according to the results of the quantitative step. ‘Error disclosure culture and Experiences with professionalism’ were the main questions in interviews to explore the participants’ experiences about the factor influencing patient safety in the COVID-19 pandemic. Based on the interview guide, and in order to increase the credibility of the interview, all of the interviews began with the following main questions: “Could you explain your experiences regarding safety challenges duration of COVID-19 epidemic?”, “Why do these challenges occur?”, “Did you experience or observe a situation that you thought endangered the patient’s safety in the clinical wards?”, “What factors do you think contributed to these challenges?”, “Could you explain your experiences regarding professionalism challenges duration of COVID-19 epidemic?” and ‘Why did the unprofessional behavior occur during the epidemic in the hospital?”, “Could you explain your experiences regarding ‘error disclosure’ challenges duration of COVID-19 epidemic?”, “Why did you or your teammates not report the error?”, In the pandemic, why you did not follow the principles of professionalism?, “What are the factors that affect the unsafe and unprofessional behavior by you and your team members during a COVID outbreak?”, Some probing questions were asked for additional clarification to the answers given by the participants. The field notes were made during the interview to reflect on the data collection process by the interviewer. The process of enrolling participants was continued until the saturation phase; that is until a complete explanation of the data was reached and no additional codes appeared.

### Analysis

All of the recorded interviews were transcribed verbatim immediately after the interview. In order to reach immersion in the data, the researcher listened to the interviews for several times and reviewed the transcripts repeatedly. Then, in order to generate the data codes, the researcher highlighted the prominent statements and freely generated data codes by taking notes in the margins of the text (open coding). At this stage, we collected the *codes* to coding sheets and generated the *categories*. We named these categories using the content-characteristic words. Finally, the *theme* emerged by comparing and contrasting of the categories. In this study, the data coding was performed by two of the authors (F.K., M.R.) and was supervised by an expert in qualitative research. In cases of disagreement over the coding, the authors would have discussed the codes until a consensus was achieved.

#### Trustworthiness

In this study, in order to establish the trustworthiness of the research, we used the criteria outlined by Schwandt et al. [19],. The *credibility* of the data was ensured through the following strategies: (a) prolonged engagement and reflection on the questions and interviews, (b) peer review (the interviews and the interpretation of data were reviewed by other researchers), and (c) member checking (data were rechecked by the participants and our interpretations of the data were rechecked and approved by them. In order to six transcripts returned to participants to obtain their comments and all of them were confirmed), (d) expert check (the analysis process and results were reviewed by two experts in the qualitative approach). In the present study, for increasing the dependability of the data, the interviews were performed uninterrupted and continued for a specific period focusing on the questions and the subject. The process of data analysis and categorization of the codes was also carefully assessed by peer researchers who were familiar with the inductive content analysis approach. We used constant comparisons to assess the semantic and structural coherence of the categories and theme. In the present study, all of the research processes such as data collection and the process of analysis were recorded in detail. We have provided a clear description of the context, the method of selecting the participants, the characteristics of the participants, the process of data collection, and the process of data analysis to facilitate the transferability of the findings.

According to the mixed methods design, the results are presented with the quantitative component as the main section, and the quantitative component is explained the influencing factors on students’ perceptions about the investigated domains of MSSAPS.

### Ethical considerations

The present study approved in the Research Ethics Committee at the National Center for Strategic Research in Medical Education (NASR) in Ministry of Health and Medical Education (ID: IR.NASRME.REC.1400.056). We have adhered to the principles of confidentiality of information and obtained informed consent for interviewing the participants, recording the interviews, and offering the rights to withdraw from the study at any time during the research. The written informed consent was obtained from all participants.

## Results

In this study, 140 medical students in the internship course participated, of which 61 (43.6%) male and 79 (56.4%) female were. The mean age of participants was 27.3 [3]. The mean grade point of the participants was 16.32 (1.33). Participants’ perception scores on safety attitude and professionalism are reported in Table 2.

**Table 2:** Participants’ perception scores on safety attitude and professionalism

	Minimum	Maximum	Mean	Std. Deviation
Safety culture	12.00	45.00	29.07	6.48
Teamwork Culture	8.00	35.00	24.94	5.23
Error disclosure culture	6.00	26.00	16.66	4.48
Experiences with professionalism	7.00	35.00	18.75	4.77
Comfort expressing professional concerns	3.00	15.00	8.59	2.96
Total	40.00	129.00	98.02	14.78

### Qualitative results

Out of 15 interns participating in the qualitative phase, 53.3% were male, 47.7% female and 60% were single. The mean age of participants was 25 years [2].

The results showed that there was no significant difference between men and women regarding the perceptions of safety attitude and professionalism (*p*-value = 0.39) and this difference was different in the domain of professional experiences (*p*-value = 0.009) (Table 3).

**Table 3:** Participants’ perception scores on safety attitude and professionalism by gender

	Gender	Mean	Std. Deviation	***p***-value
Safety culture	Male	29.57	5.66	0.42
	Female	28.68	7.01	
Teamwork Culture	Male	25.80	5.64	0.08
	Female	24.27	4.84	
Error disclosure culture	Male	16.09	4.48	0.19
	Female	17.10	4.46	
Experiences with Professionalism	Male	19.93	5.42	0.**009**
	Female	17.83	3.99	
Comfort Expressing Professional Concerns	Male	8.45	3.11	0.63
	Female	8.69	2.80	
Total	Male	99.86	15.45	0.19
	Female	96.59	14.15	

The results showed that the correlation between participants’ scores in the domain of’ medical error disclosure’ and domains of ‘safety culture’ (*r* = 0.4, *p* = 0.0001) and ‘comfort expressing professional concerns’ (*r* = 0.34, *p* = 0.0001) were positive and significant. Also, the relationship between interns’ scores in the domain of ‘comfort expressing professional concerns’ and ‘teamwork culture’ (*r* = 0.30, *p* = 0.0001) was a positive and significant relationship. According to Cohen’s index, all correlation among the scores of domains was in the moderate level [20].

### Qualitative results

**Fig. 1:**
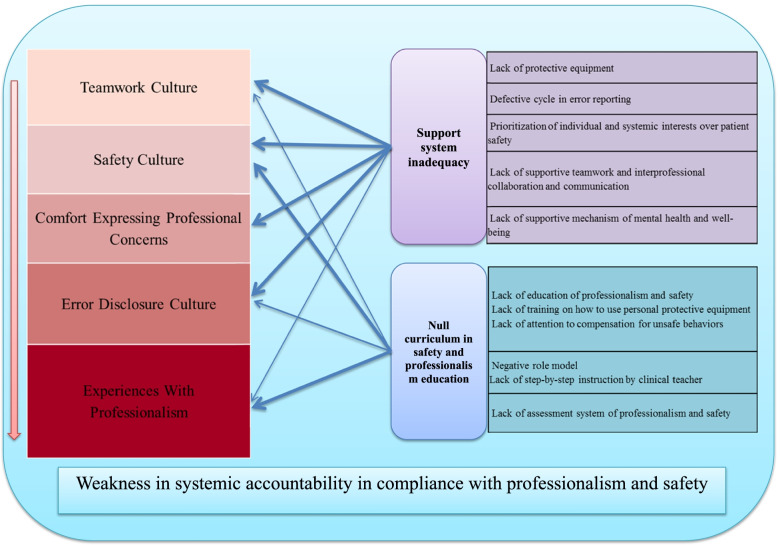
The results of the interns’ perceptions of safety attitude and professionalism and their experiences about the factors influencing on adherence of the principles during the COVID-19 pandemic

The results were explained in a theme of “weakness in systemic accountability in compliance with professionalism and safety”. The theme included two categories ‘include support system inadequacy’, and ‘null curriculum in safety and professionalism education’ (Fig. 1).

In this study, participants believed that various systemic factors have an impact on the occurrence of unsafe and unprofessional behaviors.

#### Support system inadequacy

In this category, the problems and challenges of supporting safe and professional behaviors were taken into account. The interns believed that they needed more psychological, physical, and social support systems during the epidemic, but this was neglected. The participants believed that the implementation of safety principles was supported for neither the patient nor the health personnel. According to the interns’ opinions, supportive mechanisms such as teamwork, interpersonal and inter-professional interactions, psychological support, well-being and mechanisms for reducing job stress were neglected duration epidemic. As well as, administration support mechanisms by the creation of an immersive culture for implementing professional and safety behaviors have not been considered. The challenges categorized in the weakness of the support system in the Covid-19 epidemic.

Regarding the lack of access to protective equipment for staff and patients, an intern stated:

*“Necessary equipment was not given to the students. Safety issues have not been considered because of budget constraints or health protocols have not been taken into account”. (Female, 25-years old).*

The inappropriate communication and unsupportive teamwork were mentioned as the causes of medical error. A participant stated:

*“One is afraid of communicating with a subordinate. The inappropriate communication leads to making the error because we do not have an interprofessional learning process”. (Female, 24-years old).*

Regarding the category of the lack of support for reporting errors and receiving negative feedback or punishment, a participant stated:

*“When a student makes an error, their first reaction is to mistreat or shout at the student, or punish him/her by giving an extra shift while it surely happens as he/she has not received the necessary training before doing the work”. (Male, 23-years old)*

*“Reporting an error harms the evaluation and affects one's score. When you do that the resident feels bad about you and others have a bad view of the reporter a well. So I prefer not to report”. (Female -25-years old).*

Regarding the reasons for unwillingness to report errors, an intern stated:

*“No one dares to report errors because of the prevailing stereotypes that disclose of team members’ error considers as disrespectful towards the other members”. (Female -26-years old).*

*Being afraid of the person in higher position causes that you do something you know is wrong due to fear of being questioned”. (Male -28-years old).*

Interns reported psychological concerns, lack of well-being, and lack of concentration and calmness at work as causes of making errors. An intern stated:

*“During the COVID-19 pandemic, the high number of patients and the high number of deaths have made us depressed. There was no support. We just had to keep going without being able to recover our energy”. (Female -24-years old).*

#### Null curriculum in safety and professionalism education

This category addressed “null curriculum,” as a curriculum that is “not taught and not learned.” Non-compliance with professional and safety principles is related to the lack of enough attention to the teaching and learning of safety and professionalism principles in the clinical education process. As well as, there was no formative assessment, and feedback mechanism related to the professionalism and safety fields. The interns claimed that the teaching hospitals have no formal and informal training program on the issues of professionalism and safety principles in various training courses. This was particularly evident during the COVID-19 pandemic, which generally affected clinical education. Despite the need for further training on safety principles and compliance with professionalism, this main issue has been neglected due to the priority of treatment. Regarding the weakness of formal curriculum, an intern stated:

*“No safety issues were identified for the students, for example, we never investigated unsafety issues as cases and learned from what had happened. Failure to identify critical incisions leads to unsafe processes”. (Female -25-years old).*

In this category, over-simplification and lack of understanding of the importance of adhering to patient safety principles and inattention to compensation for errors were declared unprofessional behavior. In this regard, an intern stated:

*“Many times I oversimplify the consequences of unsafe behavior and don't care about the consequences. People do not feel threatened or have the necessary knowledge about the side effects have on themselves, their families. Above all, they don't try to make amends for their errors”. (Male -27-years old)*.

In other cases, the problems of the educational system such as a large number of students with different educational objectives at the patient’s bedside and lack of proper supervision and assessment system lead to neglect the principles of safety and professionalism in clinical education. An intern stated:

*“My friends just pass the course and before they gain the necessary experience and key skills in each course, they go to the next stage of the internship and work for the patient. So they have high error rates”. (Male −27-years old)*

*“We have neither theoretical nor practical training. There is no harsh punishment for making errors and unsafe behavior. We do not have adequate training and we do not have an evaluation and feedback mechanism”. (Female -26-years old)*

One of the problems in the field of training was related to the lack of time of clinical teacher to teach professional topics, and patient safety in the epidemic period. They played their roles as a negative role model. In this regard, an intern stated:

*“In my opinion, the students should be trained under the supervision of a clinical teacher, but during the epidemic, some teachers do not have enough time and patience to teach, and the teaching situation is very rare. Instead, I have to work hard for self- learning and take the risk of making an error at work to learn”. (Female −27-years old)*

Lack of formative assessment and feedback mechanism was explained in the category as a challenges safety and professionalism. In this regard, an intern stated:

*“When you don't wear a mask or don’t disinfect your hands during Covid-19 epidemic, you and the patient may be at risk. I saw many times my friends wear not the protection equipment in the clinical wards. No one gives feedback them”. (Male −26-years old)*

Lack of assessment system and high patient load led to the interns conducted care activities without adequate competence during the epidemic. An intern stated:

*“You do something about which you do not have enough information, but since you are a medical student healthcare workers have to do something for the patient”.*

A participant stated:

*“There was no monitoring system. In the case of interns and stagers, the patient is injured due to a lack of knowledge and experience in diagnosis and treatment. In higher level, patients’ care is endangered due to fatigue and overwork. However, healthcare workers have no professional commitment to the patient and the system”. (Male -26-years old).*

## Discussion

The results indicated that the interns’ perception scores on safety attitude and professionalism are lower than moderate level. Interns’ experiences regarding the causes affecting factors were explained in a theme entitled “weakness in systemic accountability in compliance with professionalism and safety”. The theme included two categories include ‘support system inadequacy’, and ‘null curriculum in safety and professionalism education’.

Concerns about the unsafety and unprofessional behaviors such as disrespectful manners and social stigma, irresponsibility, and dishonor of patients during the Covid-19 increased [21–25]. The results showed interns’ perceptions of compliance with professionalism and safety were not appropriate. The lowest score of the interns was in the domain of ‘experiences with professionalism’ and ‘error disclosure culture’. The interns explained the weakness of systemic factors to explain the cause of the quantitative results.

The adherence of professionalism and safety principles during the COVID-19 pandemic, which is considered a critical condition for healthcare systems, is significantly important [7].

In the domain of the ‘experiences with professionalism’, issues such as understanding disrespectful behavior, saying derogatory or offensive statements about the patient, and giving inappropriate responses by the physician or residents to patient questions were addressed [14]. The lowest score were reported in the item of “my superiors behaved inappropriately, but I did not report it because I was afraid it would affect my evaluation’ in the professional experiences domain. In explaining the low scores in this domain, the participants considered a more important role for the null curriculum in safety and professionalism education. The null curriculum addressed two dimensions including an experiential null curriculum and an implemented null curriculum. The experiential null curriculum was defined as a curriculum that has been taught by teachers but not learned by the learners and the implemented null curriculum was defined as intellectual processes or contents that teachers remove or ignore. The null curriculum takes multiple dimensions, arises at hierarchical levels, and is recognized by different frames of reference (26). In the present study, the challenges of null curriculum in safety and professionalism education in both experimental and implemented levels were explored. The development of a culture of safety and the institutionalization of professional identity among students is influenced by the formal and hidden educational processes that students observe and experience in the process of clinical education [27, 28]. The students need to acquire the knowledge, and institutionalize the attitude and skills necessary to comply with professionalism and safety principles, the formal and informal education of the patient safety and professionalism must be considered from beginning, the learners enter to medical schools [29]. The present results showed that the lack of attention to the formal education and negative role models in the investigated context were effective in the scores of interns’ perception. The participants believed that the lack of time and attention to professionalism education due to the multiple roles played by clinical teachers was an important factor in the occurrence of unsafe and unprofessional behaviors. Clinical teachers were considered to as a negative role model due to non-compliance with professional principles and responsibility towards technical and professional tasks. The issue of clinical education during the COVID-19 pandemic was affected by the provision of services to COVID patients. Although, interns were required to attend clinical wards, they were less educated and focused mainly on providing services to COVID patients. The present results showed that factors such as oversimplifying the effects of unsafe behavior, performing unprofessional behaviors such as diagnostic treatment interventions without sufficient competence, and delegating authority to a person who is not competent enough due to the patient load and lack of assessment and feedback mechanism increased the unsafe behaviors. As well as, being unfamiliar with safety issues and professional principles caused the interns to experience professional and ethical dilemmas regarding the obligation to provide services in situations where they are at risk. During the COVID-19 pandemic, health workers’ fear of spreading the COVID virus prevented them from providing the best healthcare services, and compliance with professional principles decreased due to doubt, fear, and tension [1]. The weakness of the educational system in addressing the professional aspects of providing services in critical situations such as Covid-19 and the underdevelopment of professional behaviors among interns lead to a decrease their willingness and motivation to provide medical services. Education on ethical dilemmas and the development of knowledge and skills in the use of personal protective equipment and patients’ protection equipment can be effective in reducing stress and providing better services. Ates et al., showed that raising awareness of patient safety for freshmen and sophomores in Australian medical schools improves students’ attitudes towards patient safety [30].

The culture of error disclosure is important during the COVID-19 pandemic crisis and high patient load. The domain of ‘error disclosure culture’ was the second domain with the lowest scores. The lowest scores were reported in the items ‘I have received education or training on how to disclose medical errors to patients’ and ‘I am encouraged by my colleagues to disclose errors to patients/families’ in the error discloser domain. The quantitative results were explained by the inadequacy of the support system of professional behavior and patient safety approach. The interns believed that lack of a supportive atmosphere, fearing the power of residents or clinical teachers, and being afraid of lowering the score in their evaluation were some of the obstacles that prevented them from disclosing the medical error and compensation process. They also mentioned some problems of noncompliance with professional principles including negative feedback to error reporting, lack of confidentiality of the error disclosure process, negative attitude towards the error reporter, perceiving error reporting as disrespect to a higher authority, and a flawed cycle of reporting error, which harmed the error disclosure process. These challenges are categorized in the ‘inadequacy support system’. According to the present results, despite the interns’ awareness of the medical error disclosure process and its importance, they do not report errors due to technical obstacles such as not defining a specific process in error reporting and cultural obstacle factors. It also seems that in the absence of the development of an organizational culture based on professionalism such as confidentiality, responsibility, and accountability for medical error in the investigated context, the implementation of this process faced many obstacles. These factors along with the vicious process of medical error disclosure in the context can make serious risks to patient safety. In line with the present results, Martinez’s results showed that 75% of interns and residents in six medical centers in the United States were reported unprofessional behaviors. The most common reasons were not telling the truth, exposing errors, and being afraid of conflict. They have also reported a positive relationship between students’ perceptions of professionalism and patient safety [31].

In the qualitative step, participants stated the role of the support system inadequacy more important in the achieved results of the disclosure error. They believed the weakness of support systems such as medical error management, interprofessional collaboration network and the negative effects of error reporting were considered as main factors influencing the low scores of perception in the domain. Lack of support system in various aspects such as psychological health, well-being program, interprofessional cooperation networks, management supportive mechanisms, and organizational culture was increased unsafe and unprofessional behaviors in the educational hospitals. As well as, the participants believed the high work pressure, and reduced supportive communication and teamwork due to uni-professional climate accelerated the process of burnout and volition among interns during the COVID-19 pandemic. Weak teamwork and inter-professional relationships, and negative feedback are inhibitors of the development of professional and safe behaviors addressed in the present study. The growth of teamwork values as a part of a supportive system can be effective in reducing medical errors. Informal feedback in the team is very important for the development of professional and safe behaviors for the students [30]. Stubbing et al., stated the medical student suffered from the stress caused by the high expectations of various stakeholders, including physicians, patients, and nurses from medical students and it put them under pressure and they try to prove themselves as a doctor rather than a medical student [5]. Similar to the present study, it seems that high patient load and pressure to provide services during the epidemic cause excessive stress, and anxiety among interns, which impairs the well-being of students and increases the possibility of unprofessional and unsafe behavior as inappropriate treatment. Similarly, Menon identified burnout as one of the challenges to the health system in the epidemic [32]. Sperling et al., described burnout among healthcare workers were at a dangerous level and recommended the establishment of systemic support for managing their burnout [33]. The development of coping strategies as a support system has introduced as a way to maintain and improve the quality of health care [32, 34]. Consistent with the results of Robert’s study, it emphasizes the development of well-being strategies for health workers and patients during the coronavirus epidemic [35]. Ćurković emphasized the use of wellness–well-being mechanisms in educational systems to develop professionalism [7]. In this regard, establishing support mechanisms for the development of well-being and the growth of professional values in health personnel as a way to improve patient safety was suggested [36]. It seems that during the epidemic, the need to establish support systems for the development of professional behaviors in line with the safety of personnel, especially novice workers and patients has increased. As well as, null curriculum explored as a challenge in this domain. Armitage et al., identified the weakness of training as one of the error disclosure problems during the COVID-19 pandemic [37] that is in line with the present study. Lee et al., explained that many medical students spoke very harshly about error disclosure. They recommended developing a guide for error disclosure and training to develop complex communication skills in difficult situations [38].

The results showed the scores of interns in the ‘comfort expressing professional concerns’ and ‘safety culture’ in blow the moderate level and ‘teamwork culture’ scores in the moderate level. Participants considered the factors resulting from both the explored themes to be important in relation to the low scores of safety culture. They believed that the lack of support system and the lack of formal education process and hidden curriculum contributed to their weak attitude in the domain of safety culture. The role of the inadequacy of support system in the scores in the domain of ‘teamwork culture’ and the ‘comfort expressing professional concerns’ were explained in viewpoints’ of the participants in the qualitative phase. The results of this study indicated a positive relationship between interns’ perception scores in the domain of ‘error disclosure culture’ and their scores in the domains of ‘safety culture’ and ‘comfort expressing professional concern’. There was also a positive and significant correlation between the interns’ scores in the domain of ‘comfort expressing professional concerns’ with ‘teamwork culture’. It can conclude, the creation of a positive interprofessional relationship facilitates the error disclosure process and compensates for the error. The present results are consistent with the results of Lee’s study [38]. The results of Liao’s study are consistent with the present results, which showed a significant relationship between medical students’ perceptions of patient safety and cultural components such as teamwork culture and culture of error disclosure [14]. Establishing inter-profession teamwork relationships and developing a safety culture in educational systems can be effective in expressing professional concerns through effective communication among healthcare workers. Therefore, creating inter-professional teams and developing an interprofessional learning process can be effective in constructing teamwork and developing effective interprofessional professionalism and safety.

In this study, female students reported lower scores on professionalism than male students. Since the mentioned questions in this domain have assessed the conduct of unprofessional behavior by theirs and other team members, female interns seem to have answered these questions conservatively (less assertiveness). In the qualitative phase, different experiences related to gender and unsafe and unprofessional behaviors were explored. Some interns believed that female interns’ conservatism made them more sensitive to unprofessional behaviors and adherence of health protocols and safety principles, leading to less unsafe behaviors. However, some interns stated that stress and lower self-esteem, obsession, and more emotional involvement of female interns lead to more unsafe behaviors. On the other hand, some unsafe behaviors were considered more common among male interns, and less sensitivity, lack of adherence to health and safety protocols, and false self-esteem were categorized among the causes of such behaviors. Similar to the present qualitative results, Colet’s results showed that women are usually more sensitive in terms of safety, quality of patient care, and the application of safety principles [39]. Brasaite et al., indicated that the positive nursing attitudes were higher in female nurses than in men [40]. Carney et al., also found that positive safety attitudes were significantly higher in female operating room nurses rather than in men [41], which differed from the present results. The difference in results was attributed to differences in the field under study (medicine versus nursing), staff work experience compared to interns, environment, and organizational culture in the studies.

### Limitations

The present study was conducted during the Covid-19 pandemic. The results of the qualitative section showed that low perceptions of patient safety and professionalism were rooted in systemic challenges more than epidemics features. Future studies were recommended to compare the safety and professionalism challenges and their causes before and after covid-19. The number of samples and self-report as well as the use of qualitative methods were performed in the teaching hospitals of a province, which can decrease the generalizability of the findings.

## Conclusion

The present results showed participants’ perception scores on safety attitude and professionalism were below the moderate level. The systemic issues were explored as influencing factors in the occurrence of unsafe and unprofessional behaviors. They reported the support system inadequacy (individual, teamwork, mental health and well-being, management, and culture), disregard for professional, and safety principles in the formal and non-formal education system effective on unprofessional and unsafe. The scores of ‘experiences with professionalism’ were the lowest level in the study. The participants acknowledged the explored themes effect on their perception of professionalism. They addressed the factors associated with null curriculum in safety and professionalism education more effective on the low scores of perception of professionalism. The domain of ‘error disclosure culture’ was the second domain with the lowest scores. Participants in this domain considered the role of the support system more important. They believed the weakness of support systems such as medical error management and the negative effects of error reporting were considered as main factors influencing the low scores of perception. They mentioned the role of education including negative role models and the lack of a teaching-learning process intended a slighter contribution to the scores of the domain. In the domain of ​​ comfort expressing professional concerns, students cited the weakness of the support system as the only perceived challenge. In the domain of safety culture, the students’ scores were below the moderate level and the two themes were explained as main challenges. Students attributed the challenges in the domain of teamwork culture due to the weakness of the support system. During the COVID-19 pandemic, it is recommended to create mechanisms to support the development of professionalism of healthcare workers, especially, novice providers and students, and pay attention to the safety and professionalism in formal and informal educational programs.

## Data Availability

The datasets used and/or analyzed during the current study are available from the corresponding author on reasonable request.
